# Renoprotective effects of a dipeptidyl peptidase 4 inhibitor in a mouse model of progressive renal fibrosis

**DOI:** 10.1080/0886022X.2017.1279553

**Published:** 2017-01-24

**Authors:** Takahiro Uchida, Takashi Oda, Hidehito Matsubara, Atsushi Watanabe, Hanako Takechi, Naoki Oshima, Yutaka Sakurai, Hiroo Kumagai

**Affiliations:** a Department of Nephrology and Endocrinology, National Defense Medical College, Tokorozawa, Saitama, Japan;; b Department of Nephrology, Tokyo Medical University Hachioji Medical Center, Hachioji, Tokyo, Japan;; c Department of Preventive Medicine and Public Health, National Defense Medical College, Tokorozawa, Saitama, Japan

**Keywords:** Chronic kidney disease, dipeptidyl peptidase 4, renal fibrosis, renal inflammation, unilateral ureteral obstruction

## Abstract

Although the effects of dipeptidyl peptidase 4 (DPP-4) inhibitors beyond their hypoglycemic action have been reported, whether these inhibitors have renoprotective effects in nondiabetic chronic kidney disease (CKD) is unclear. We examined the therapeutic effects of DPP-4 inhibition in mice with unilateral ureteral obstruction (UUO), a nondiabetic model of progressive renal fibrosis. After UUO surgery, mice were administered either the DPP-4 inhibitor alogliptin or a vehicle by oral gavage once a day for 10 days. Physiological parameters, degrees of renal fibrosis and inflammation, and molecules related to renal fibrosis and inflammation were then evaluated using sham-operated mice as controls. Positive area of α-smooth muscle actin was significantly smaller and expression of transforming growth factor β messenger RNA was significantly lower in the alogliptin-treated group than in the vehicle-treated group. Renal total collagen content was also significantly lower in the alogliptin-treated group than in the vehicle-treated group. These results suggest that alogliptin exerted renoprotective antifibrotic effects. The positive area of F4/80 was significantly smaller and expression of CD68 messenger RNA was significantly lower in the alogliptin-treated group than in the vehicle-treated group, suggesting an anti-inflammatory action by the DPP-4 inhibitor. Compared to the results for the vehicle-treated group, expression of markers for M1 macrophages tended to be lower in the alogliptin-treated group, and the relative expression of M2 macrophages tended to be higher. These data indicate the various protective effects of DPP-4 inhibition in nondiabetic mice with UUO. DPP-4 inhibitors may therefore be promising therapeutic choices even for nondiabetic CKD patients.

## Introduction

Since the incidence of chronic kidney disease (CKD) has been increasing and CKD has become a worldwide medical and socioeconomic problem, strategies to improve CKD outcomes are needed.^1^ At present, the only medications known to be effective in the treatment of CKD patients are renin-angiotensin system (RAS) inhibitors: angiotensin-converting enzyme (ACE) inhibitor and angiotensin II type 1 receptor blocker (ARB).^2^^,^^3^ Despite the significant renoprotective effect of RAS inhibitors, many CKD patients treated with RAS inhibitors progress to end-stage renal failure. Furthermore, RAS inhibitors typically cannot be administered to patients with hyperkalemia or those who are hypotensive, leading to an urgent need for alternative therapeutic strategies.

Dipeptidyl peptidase 4 (DPP-4) is an exopeptidase, which removes N-terminal dipeptides from polypeptide chains. Many compounds have been identified as DPP-4 substrates, and glucagon-like peptide 1 (GLP-1), an incretin hormone important for postprandial glycemic control and regulation of satiety, is known as an important DPP-4 substrate.^4^ DPP-4 inhibitors are therefore widely used in the treatment of type 2 diabetes mellitus.

In addition to hypoglycemic effects, DPP-4 inhibitors also exert antifibrotic effects. DPP-4 inhibition was reported to suppress transforming growth factor β (TGF-β) and α-smooth muscle actin (α-SMA) expression,^5^ and treatment with a DPP-4 inhibitor reduced fibrosis in a model of myocarditis in mice.^6^ Anti-inflammatory effects have also been suggested. Improvement of atherosclerosis by DPP-4 inhibition presumably due to inhibition of monocyte activation/chemotaxis has been reported.^7^ DPP-4 inhibition has also been reported to prevent the infiltration of proinflammatory macrophages of the M1 phenotype.^8^

Studies evaluating the pleiotropic effects of DPP-4 inhibitors in CKD, especially in nondiabetic CKD, have been scarce.^9^ In the present study, we examined the therapeutic effects of DPP-4 inhibition in mice with unilateral ureteral obstruction (UUO), a nondiabetic model of progressive renal fibrosis.

## Materials and methods

### Animal model and experimental protocol

Male C57BL/6J mice 6- to 8-week-old were obtained from CLEA Japan (Tokyo, Japan) and provided water and standard chow *ad libitum*. All animal experiments were conducted in accordance with the National Defense Medical College guidelines for the care and use of laboratory animals in research, and the study protocol (no. 14009) was approved by the Animal Ethical Committee of the National Defense Medical College.

Mice were randomly assigned to three groups: (1) An alogliptin-treated group (*n* = 9), which received 40 mg/kg of alogliptin (Takeda Pharmaceutical Co. Ltd., Tokyo, Japan)^7^ by oral gavage once a day for 10 days after UUO surgery; (2) a vehicle-treated group (*n* = 8), which received saline by oral gavage once a day for 10 days after UUO surgery; (3) a sham-operated group (*n* = 10). The UUO surgery was performed as described by Yamada et al.^10^

Systolic blood pressure (SBP) was measured by tail-cuff plethysmography before the UUO surgery and at the end of the experiment. Mice were sacrificed under deep anesthesia induced by an intraperitoneal injection of sodium pentobarbital. The left kidneys (the UUO-operated kidneys) were harvested and cut into several pieces that were either fixed in 10% formalin and embedded in paraffin for histological analysis or stored at −80 °C for subsequent studies.

### Physiological parameters

Blood urea nitrogen (BUN), serum creatinine, and total cholesterol at the end of the experiment were measured by a clinical laboratory testing company (SRL, Tokyo, Japan) by standard methods using an automatic analyzer. Tail blood glucose was measured using a blood glucose meter (Sanwa Kagaku, Nagoya, Japan).

### Renal histopathology

Assessment of renal fibrosis: Renal fibrosis level was evaluated histologically by picrosirius red staining of sections of paraffin-embedded tissue (3 μm thick) as described by Oda et al.^11^

Immunofluorescence (IF) staining: Indirect IF staining for α-SMA (myofibroblast marker), F4/80 (monocytes/macrophage marker), or phosphorylated (p)-Smad 3 was performed on 5-μm cryostat sections of fresh frozen kidney. After fixation and blocking with blocking buffer, sections were incubated with mouse FITC-conjugated anti-α-SMA antibody (Sigma-Aldrich, St. Louis, MO)^10^, rat anti-F4/80 antibody (Serotec Ltd., Oxford, UK), or anti-p-Smad 3 antibody (Abcam, Cambridge, UK). The bound antibodies were visualized by incubating the sections with goat anti-FITC antibody (GeneTex, Irvine, CA), Alexa Fluor (AF) 488-conjugated donkey anti-rat antibody, or AF 488-conjugated donkey anti-rabbit antibody (Thermo Fisher Scientific, Waltham, MA). The sections were then counterstained with Hoechst 33342 (Thermo Fisher Scientific) for nuclear staining.

Calculation: Images of five nonoverlapping tubulointerstitial fields from each section were obtained with a digital camera at ×200 magnification. The percentages of the tubulointerstitial areas positive for picrosirius red, α-SMA, or F4/80 staining were measured with image analysis software (LuminaVision ver. 2.04, Mitani Corporation, Tokyo, Japan), and p-Smad 3-positive cells in the tubulointerstitial area were counted. The percentages and numbers were averaged in each group.

### Renal total collagen content

Renal total collagen content was evaluated using a simple quantitative microassay kit for collagen and noncollagenous proteins (Chondrex Inc., Redmond, WA)^12^ in accordance with the manufacturer’s instructions. In brief, paraffin-embedded tissue sections were stained with Sirius Red and Fast Green for collagen and noncollagen protein. The dyes were then extracted and measured at optical density (OD) 540 (Sirius Red) and OD 605 (Fast Green). The relative collagen content was calculated and expressed as micrograms per milligram protein.

### Real-time reverse transcription-polymerase chain reaction (RT-PCR)

Extraction of total RNA, reverse transcription into complementary DNA, and subsequent real-time PCR were all performed as described by Takechi et al.^13^ We used primer/probe sets of TaqMan Gene Expression Assays for mouse TGF-β, collagen I, plasminogen activator inhibitor 1 (PAI-1), CD68, CD11c, inducible nitric oxide synthase (iNOS), CD206, GLP-1 receptor (GLP-1R), DPP-4, and glyceraldehyde 3-phosphate dehydrogenase (GAPDH), all obtained from Thermo Fisher Scientific. CD11c^8^ and iNOS have been reported to be preferentially expressed in M1 macrophages, whereas CD206 has been reported to be preferentially expressed in M2 macrophages. The relative amount of messenger RNA (mRNA) was calculated using the comparative Ct (ΔΔCt) method. All amplification products were normalized against GAPDH mRNA, which was amplified in the same reaction as an internal control.

### Protein extraction

Protein samples for the assessment of DPP-4 activity in renal tissue were extracted in a lysis buffer of cold PBS (3 μL per 1 mg of renal tissue) containing 1% Triton X-100 and 1 K/IU/mL of aprotinin,^14^ while those for the assessment of other proteins were extracted as described by Kushiyama et al.^15^ Protein concentrations were determined using BCA protein assay kits (Pierce, Rockford, IL).

### Assay for DPP-4 activity and active GLP-1

Serum and renal DPP-4 activity was assessed as described by Moritoh et al.^16^ using 10 μL of serum or renal homogenates containing 140 μg of protein.

Renal GLP-1 levels were measured using a fluorescence enzyme-linked immunosorbent assay (ELISA) kit (Glucagon-Like Peptide-1 (Active) ELISA kit, Millipore, St. Charles, MO). All measurement procedures were performed in accordance with the manufacturer’s instructions, with one exception; we used an excitation wavelength of 330 nm instead of 355 nm.

### ELISA for adiponectin

Serum and renal adiponectin levels were measured using a mouse/rat adiponectin ELISA kit (Otsuka Pharmaceutical Co., Tokyo, Japan) in accordance with the manufacturer’s instructions.

### Statistical analysis

Data are expressed as the mean ± SE. Differences between two experimental groups were assessed using Student’s *t*-test, and those among the three experimental groups were assessed by one-way ANOVA with *post hoc* analysis. *p* Values lower than 0.05 were considered significant. All statistical analyses were performed with JMP10 statistics software (SAS Institute Inc., Cary, NC).

## Results

### Physiological parameters

SBP, body weight, serum parameters, and blood glucose levels are shown in Table 1. BUN and serum creatinine levels in the vehicle-treated group were significantly higher than those in the sham-operated group, but the levels tended to be lower in the alogliptin-treated group. No significant differences were observed between the alogliptin-treated and the vehicle-treated groups in terms of SBP, body weight, blood glucose, or serum total cholesterol. Although the differences were not statistically significant, both serum and renal adiponectin levels in the alogliptin-treated group tended to be higher than those in the vehicle-treated group (Table 1).

**Table 1.** Physiological parameters at the end of the experiment.

	Sham	Vehicle	Alogliptin
Number of mice	10	8	9
Systolic BP (mmHg)	Not tested	101.78 ± 1.95	99.00 ± 1.70
Body weight (g)	20.71 ± 0.58	19.93 ± 0.94	20.39 ± 0.86
Blood urea nitrogen (mg/dL)	26.07 ± 1.77	36.96 ± 3.80^a^	33.53 ± 3.06
Serum creatinine (mg/dL)	0.127 ± 0.017	0.183 ± 0.020^a^	0.179 ± 0.020
Blood glucose (mg/dL)	83.67 ± 9.40	92.88 ± 8.12	96.00 ± 6.62
Total cholesterol (mg/dL)	71.10 ± 4.34	79.38 ± 2.97	82.63 ± 7.45
Serum adiponectin (μg/mL)	36.62 ± 3.34	33.72 ± 6.82	41.20 ± 8.37
Renal adiponectin (mg/g protein)	5.74 ± 0.63	5.72 ± 1.24	7.60 ± 1.03

Data are shown as mean ± SE.

a *p*< 0.05 compared with the sham-operated group.

### Degree of renal fibrosis and renal expression of profibrotic molecules

**Figure 1.**
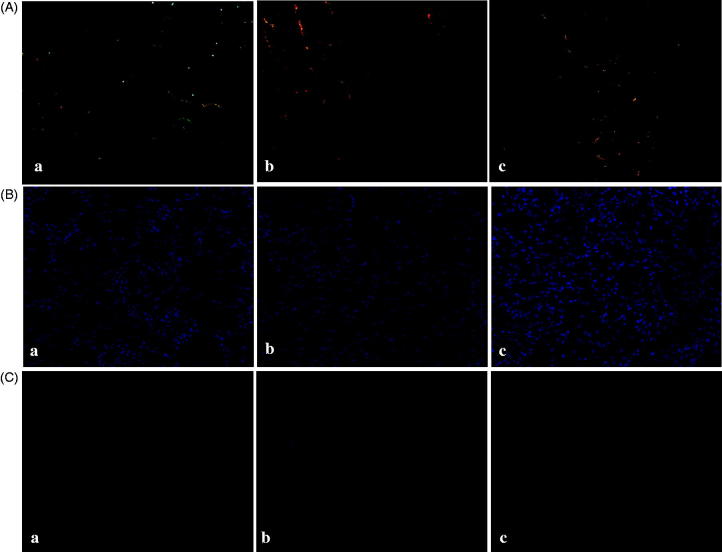
Representative images of (A) picrosirius red-stained, (B) α-smooth muscle actin-stained, and (C) phosphorylated Smad 3-stained slices of kidneys from mice in the (**a**) sham-operated, (**b**) vehicle-treated, and (**c**) alogliptin-treated groups (original magnification in all panels, ×200).

**Figure 2.**
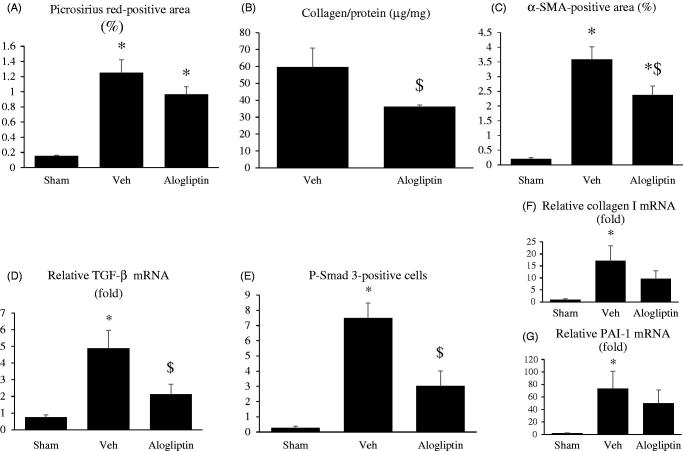
(A) Percentage of tubulointerstitial area positive for picrosirius red staining in kidneys in the sham-operated (sham, *n* = 10), vehicle-treated (veh, *n* = 8), and alogliptin-treated (alogliptin, *n* = 9) groups. *p < 0.001 compared with the sham-operated group. (B) Renal total collagen content in the vehicle-treated and alogliptin-treated groups. $*p* < 0.05 compared with the vehicle-treated group. (C) Percentage of tubulointerstitial area positive for α-smooth muscle actin (α-SMA) staining in kidneys in each group. *p < 0.001 compared with the sham-operated group. $*p* < 0.01 compared with the vehicle-treated group. (D) Relative renal expression levels of transforming growth factor β (TGF-β) messenger RNA (mRNA) in each group. Glyceraldehyde 3-phosphate dehydrogenase (GAPDH) mRNA was used as the internal control to adjust for unequal total mRNA content. *p < 0.001 compared with the sham-operated group; $*p* < 0.01 compared with the vehicle-treated group. (E) Number of tubulointerstitial cells positive for phosphorylated Smad 3 (p-Smad 3) staining in kidneys in each group. *p < 0.001 compared with the sham-operated group. $*p* < 0.01 compared with the vehicle-treated group. Relative expression levels of (F) collagen I mRNA and (G) plasminogen activator inhibitor 1 (PAI-1) mRNA in kidneys in each group. GAPDH mRNA was used as the internal control to adjust for unequal total mRNA content. **F** *p < 0.01 compared with the sham-operated group. **G** *p < 0.05 compared with the sham-operated group.

Figure 1(A) shows representative images of picrosirius red staining in the sham-operated (a), vehicle-treated (b), and alogliptin-treated (c) groups. The proportion of picrosirius red staining-positive interstitial area was larger in the vehicle-treated group than in the sham-operated group and tended to be smaller in the alogliptin-treated group. However, the difference between the vehicle-treated and the alogliptin-treated groups did not reach statistical significance (*p* = 0.08, Figure 2(A)). Renal total collagen content in the alogliptin-treated group was significantly lower than that in the vehicle-treated group (Figure 2(B)).

Figure 1(B) shows representative images of α-SMA staining in the sham-operated (a), vehicle-treated (b), and alogliptin-treated (c) groups. The α-SMA staining-positive interstitial area was larger in the vehicle-treated group than in the sham-operated group, and significantly smaller in the alogliptin-treated group than in the vehicle-treated group (Figure 2(C)).

Real-time RT-PCR revealed that the expression of TGF-β mRNA in the alogliptin-treated group was significantly lower than that in the vehicle-treated group (Figure 2(D)). The number of p-Smad 3-positive cells was greater in the vehicle-treated group than in the sham-operated group, and significantly smaller in the alogliptin-treated group than in the vehicle-treated group (Figure 1(C) and 2(E)).

The expression of collagen I and PAI-1 mRNA tended to be lower in the alogliptin-treated group than in the vehicle-treated group. However, the differences between the alogliptin-treated and vehicle-treated groups were not statistically significant (Figure 2(F,G)).

### Degree of macrophage infiltration, expression of proinflammatory molecules, and macrophage phenotypes

**Figure 3.**
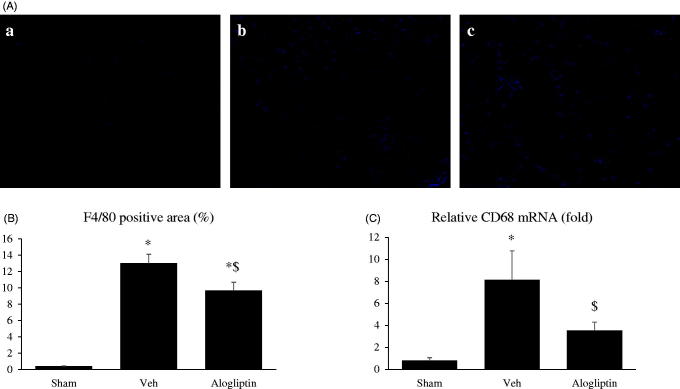
(A) Representative images of F4/80-stained slices of kidneys from mice of the (**a**) sham-operated, (**b**) vehicle-treated, and (**c**) alogliptin-treated groups (original magnification, ×200). (B) Percentage of tubulointerstitial area positive for F4/80 staining in kidneys in the sham-operated (sham, *n* = 10), vehicle-treated (veh, *n* = 8), and alogliptin-treated (alogliptin, *n* = 9) groups. *p < 0.001 compared with the sham-operated group. $*p* < 0.01 compared with the vehicle-treated group. (C) Relative expression levels of CD68 messenger RNA (mRNA) in kidneys in each group. Glyceraldehyde 3-phosphate dehydrogenase (GAPDH) mRNA was used as the internal control to adjust for unequal total mRNA content. **C** *p < 0.01 compared with the sham-operated group; $*p* < 0.05 compared with the vehicle-treated group.

Figure 3(A) shows representative images of F4/80 staining in the sham-operated (a), vehicle-treated (b), and alogliptin-treated (c) groups. Prominent interstitial infiltration of macrophages was seen in the vehicle-treated group, and this interstitial macrophage infiltration was significantly milder in the alogliptin-treated group (Figure 3(B)).

Results for real-time RT-PCR to assess mRNA expression of proinflammatory molecules were consistent with the findings from F4/80 staining. The expression of CD68 mRNA was significantly lower in the alogliptin-treated group than in the vehicle-treated group (Figure 3(C)).

**Figure 4.**
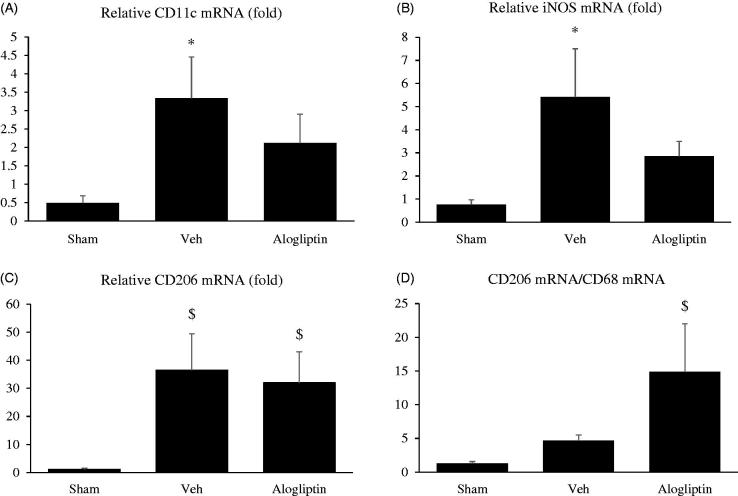
Relative expression levels of (A) CD11c messenger RNA (mRNA), (B) inducible nitric oxide synthase (iNOS) mRNA, and (C) CD206 mRNA in the kidneys in the sham-operated (sham, *n* = 10), vehicle-treated (veh, *n* =8), and alogliptin-treated (alogliptin, *n* = 9) groups. Glyceraldehyde 3-phosphate dehydrogenase (GAPDH) mRNA was used as the internal control to adjust for unequal total mRNA content. **A**, **B** *p <0.01 compared with the sham-operated group. **C** $*p* < 0.05 compared with the sham-operated group. (D) Ratio of CD206 mRNA to CD68 mRNA in each group. $*p* < 0.05 compared with the sham-operated group.

To evaluate the phenotypic change in macrophages between the experimental groups, we analyzed the mRNA levels of CD11c, iNOS, and CD206. Although the differences were not statistically significant, the expression levels of both CD11c mRNA (Figure 4(A)) and iNOS mRNA (Figure 4(B)) in the alogliptin-treated group tended to be lower than those in the vehicle-treated group. In contrast to the mildness of total macrophage infiltration in the alogliptin-treated group, CD206 mRNA expression did not differ between the vehicle-treated and alogliptin-treated groups (Figure 4(C)). In addition, although the difference did not reach statistical significance (*p* = 0.10), the relative expression of M2 macrophages, assessed by the ratio of CD206 mRNA levels to CD68 mRNA levels,^17^ tended to be higher in the alogliptin-treated group (Figure 4(D)).

### DPP-4 activity, GLP-1 levels, and DPP-4 and GLP-1R mRNA levels

Serum and renal DPP-4 activity, renal GLP-1 levels, and DPP-4 and GLP-1R mRNA expression levels are shown in Table 2. Serum DPP-4 activity in the alogliptin-treated group tended to be lower than that in the vehicle-treated group, but the difference did not reach statistical significance. In contrast, renal DPP-4 activity in the alogliptin-treated group was significantly lower than that in the vehicle-treated group. Contrary to the findings for renal DPP-4 activity, the expression of renal DPP-4 mRNA in the UUO-operated groups tended to be higher than that in the sham-operated group. Although the mean renal GLP-1 level of the alogliptin-treated group was more than twice that of the vehicle-operated group, the difference was not statistically significant. Unlike the findings reported by Min et al.,^9^ there was no significant difference in the expression of GLP-1R mRNA between the UUO-operated groups and the sham-operated group.

**Table 2.** Serum and renal DPP-4 activity, renal GLP-1 level, and level of renal DPP-4 and GLP-1R mRNA.

	Sham	Vehicle	Alogliptin
Serum DPP-4 activity	0.082 ± 0.011	0.073 ± 0.016	0.063 ± 0.011
Renal DPP-4 activity	0.454 ± 0.023	0.236 ± 0.011^a^	0.146 ± 0.003^a^^,^^b^
Renal GLP-1 (pM)	13.39 ± 2.13	10.30 ± 2.905	21.21 ± 12.18
Relative DPP-4 mRNA (fold)	0.79 ± 0.27	3.46 ± 1.90	2.62 ± 0.96
Relative GLP-1R mRNA (fold)	1.37 ± 0.41	0.90 ± 0.21	0.74 ± 0.13

Data are shown as mean ± SE.

a *p*< 0.01 compared with the sham-operated group.

b *p*< 0.01 compared with the vehicle-treated group.

### Correlation between DPP-4 activity and renal adiponectin levels or PAI-1 mRNA expression levels

**Figure 5.**
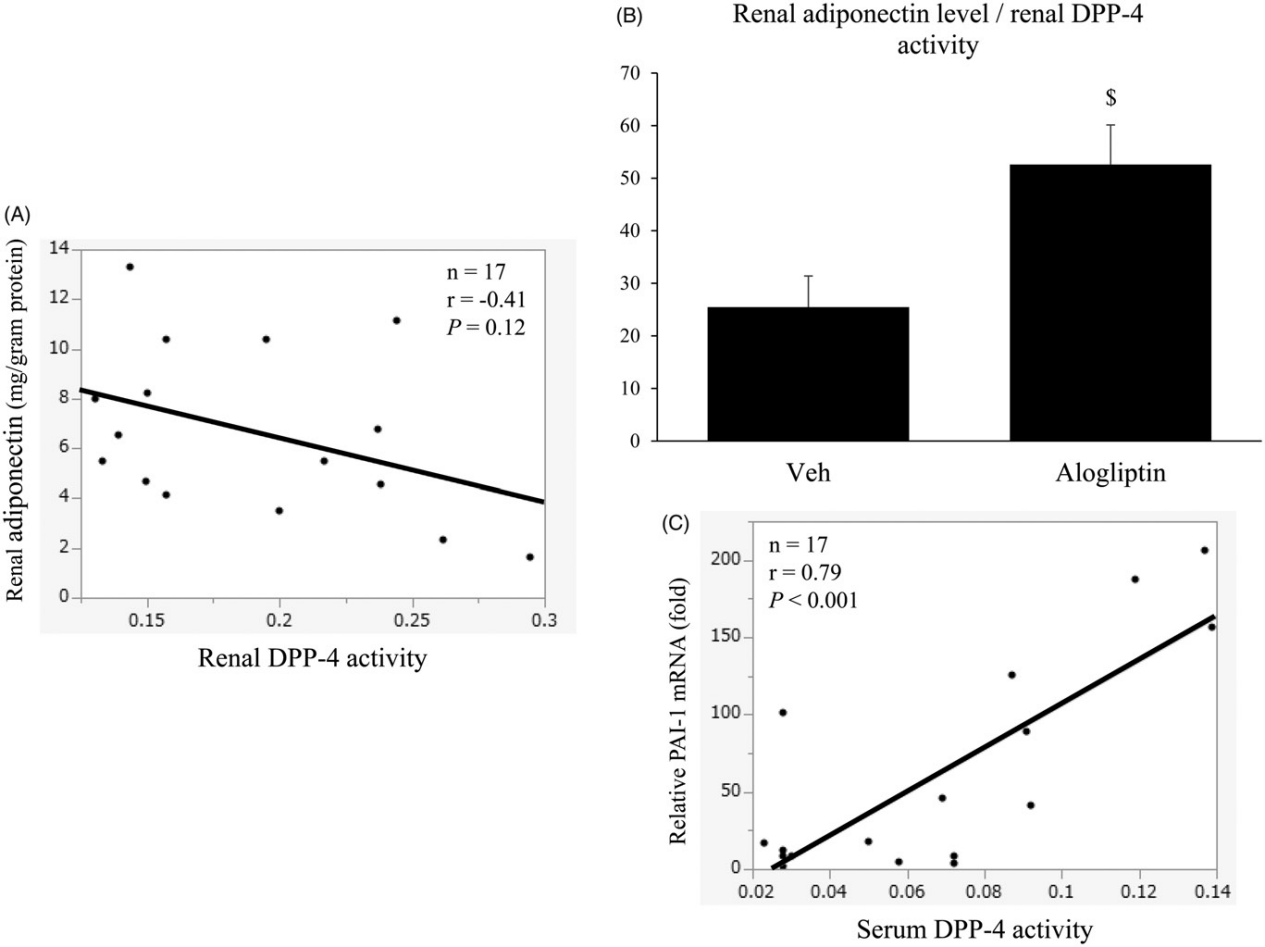
(A) Correlation between renal dipeptidyl peptidase 4 (DPP-4) activity and renal adiponectin levels in the vehicle-treated and alogliptin-treated groups. (B) Ratio of renal adiponectin levels to renal DPP-4 activity in the vehicle-treated (veh, *n* = 8) and alogliptin-treated (alogliptin, *n* = 9) groups. ^$^*p* < 0.05 compared with the vehicle-treated group. (C) Correlation between serum DPP-4 activity and relative renal expression levels of plasminogen activator inhibitor 1 (PAI-1) mRNA in the vehicle-treated and alogliptin-treated groups.

We further evaluated the correlation between renal DPP-4 activity and renal adiponectin levels or PAI-1 mRNA expression levels in the UUO-operated groups. Figure 5(A) shows an apparent negative correlation between renal DPP-4 activity and renal adiponectin levels (*r*= −0.41), but the correlation was not significant (*p*= 0.12). However, the ratio of renal adiponectin levels to renal DPP-4 activity in the alogliptin-treated group was significantly higher than that in the vehicle-treated group (*p* < 0.05, Figure 5(B)). Serum DPP-4 activity was significantly and positively correlated with PAI-1 mRNA expressions levels (*r* = 0.79, *p*< 0.001, Figure 5(C)).

## Discussion

The mechanisms of antifibrotic and anti-inflammatory effects seen in this study were clearly irrelevant to the hypoglycemic effect of DPP-4 inhibitors or their reported effect on BP^18^ because there was no significant difference in blood glucose or SBP between the alogliptin-treated and vehicle-treated groups.

We believe that the renoprotective action of alogliptin in the present study resulted from its inhibition of tubulointerstitial fibrosis. Interstitial expression of α-SMA, which shows the presence of interstitial myofibroblasts, was significantly lower in the alogliptin-treated group than that in the vehicle-treated group, and renal total collagen content was significantly lower. Although the differences did not reach statistical significance, the tubulointerstitial fibrotic area assessed by picrosirius red staining tended to be smaller, and the expression levels of collagen I and PAI-1 mRNA tended to be lower in the alogliptin-treated group than in the vehicle-treated group. We found significantly lower expression of TGF-β mRNA in the alogliptin-treated group, which we suspect is the molecular mechanism responsible for these antifibrotic effects. Reduction of cardiac fibrosis by a DPP-4 inhibitor has been reported.^6^ DPP-4 inhibition has been shown to suppress TGF-β and α-SMA expression in renal tubular epithelial cells by blocking NF-κB,^5^ and attenuation of NF-κB and TGF-β signaling through DPP-4 inhibition has also been reported to suppress renal fibrosis.^9^ We also showed that the number of p-Smad 3-positive cells was significantly lower in the alogliptin-treated group than in the vehicle-treated group, thereby suggesting that DPP-4 inhibition suppressed downstream pathway of the TGF-β signaling.

Another factor that may contribute to the protective effects of DPP-4 inhibition is its anti-inflammatory effect through inhibition of macrophage infiltration. Interstitial infiltration of macrophages, as assessed by F4/80 staining, was significantly milder in the alogliptin-treated group than in the vehicle-treated group, and CD68 mRNA expression was significantly lower. In accordance with these findings, DPP-4 inhibition was reported to inhibit monocyte activation/chemotaxis and thereby lead to the improvement of atherosclerosis in a mouse model of atherosclerosis and insulin resistance.^7^ In addition, although our results did not reach statistical significance, the expression of proinflammatory M1 macrophage markers (the expression of CD11c and iNOS mRNA by real-time PCR) tended to be lower, and the relative expression of anti-inflammatory M2 macrophages (the ratio of CD206 mRNA levels to CD68 mRNA levels) tended to be higher in the alogliptin-treated group than in the vehicle-treated group. These tendencies in the shift in macrophage polarization may also modulate the anti-inflammatory effects of alogliptin seen in the present study.

Upregulated expression of renal DPP-4 by stimulation with inflammatory cytokines and reduced production of inflammatory cytokines by mononuclear cells through DPP-4 inhibition have also been reported.^19^ Thus, DPP-4 inhibition appears to exert its anti-inflammatory effects in part by blocking the cycle of inflammation. We selected the alogliptin dose (40 mg/kg daily) according to a previous study,^7^ in which serum DPP-4 activity was significantly suppressed. However, that dose suppressed renal DPP-4 activity but not serum DPP-4 activity in the present study. A difference in the animal models used may account for the discrepancy. We think, however, that the decreased DPP-4 activity we found in the kidney but not in the blood is important because active inflammation occurred locally in the UUO-operated kidney. On the other hand, our finding that renal DPP-4 activity in the sham-operated group was higher than that in the vehicle-treated group was unexpected, because severe inflammation was found only in the vehicle-treated group. There was also some discrepancy between DPP-4 enzymatic activity and DPP-4 mRNA levels in the present study (Table 2). The precise mechanism is yet to be identified; however, it appears possible that renal DPP-4 activity does indeed decrease in some models of renal diseases. A similar condition (reduced renal DPP-4 activity despite enhanced protein levels) has been reported in a rat model of diabetic nephropathy.^20^

DPP-4 itself has been reported to be an adipokine that impairs insulin sensitivity, and serum DPP-4 levels have been shown to be negatively correlated with serum adiponectin levels.^21^ It has also been reported that DPP-4 inhibitors can cause an increase in plasma adiponectin levels.^22^ In the present study, we compared DPP-4 activity and adiponectin levels between alogliptin-treated and vehicle-treated UUO-operated kidneys, and showed that renal adiponectin levels in the alogliptin-treated group tended to be higher than those in the vehicle-treated group. In addition, the ratio of renal adiponectin levels to renal DPP-4 activity in the alogliptin-treated group was significantly higher than that in the vehicle-treated group. This finding may suggest that local adiponectin levels increase in response to inhibition of local DPP-4 activity. We also showed that serum DPP-4 activity was significantly and positively correlated with PAI-1 mRNA expression levels.

Unlike the findings reported by Min et al.,^9^ there was no significant difference in GLP-1R mRNA expression levels between the UUO-operated groups and the sham-operated group in the present study. In addition, renal GLP-1 levels in the alogliptin-treated group tended to be higher than those in the vehicle-treated group. We did not evaluate GLP-1 mRNA levels in renal tissue, and therefore, the possibility of local GLP-1 generation in the kidney could not be ruled out. However, we think that renal GLP-1 is not generated *in situ* but rather derived from circulating blood, because GLP-1 is an incretin hormone, which is generated in the gastrointestinal tract. In renal tissue, DPP-4 is reportedly expressed on proximal tubules and glomerular podocytes, but the expression pattern of GLP-1R is unclear: In human renal tissue, it was reported to be expressed predominantly in proximal tubules, while in mouse renal tissue, it was reported to be localized in glomerular capillary walls and throughout vascular walls.^23^ Thus, GLP-1R may exhibit different expression patterns in different species. However, it is reasonable to suppose that DPP-4 inhibition activates GLP-1 signaling in renal tissue. It has been shown that GLP-1 exerts several effects other than hypoglycemic action, such as anti-inflammatory effects^24^ and antiapoptotic effects.^25^ In addition, its effects are reportedly independent of DPP-4 inhibition; some studies have reported that GLP-1 mimetics could induce antifibrotic effects.^26^^,^^27^

In summary, DPP-4 inhibition suppressed renal fibrosis and inflammation in a nondiabetic model of progressive renal fibrosis in mice, suggesting a renoprotective role. DPP-4 inhibitors may therefore be promising therapeutic choices for CKD patients even if they are nondiabetic.
